# The prognostic impact of *RAS* on overall survival following liver resection in early versus late-onset colorectal cancer patients

**DOI:** 10.1038/s41416-020-01169-w

**Published:** 2020-11-19

**Authors:** Alexandre A. Jácome, Timothy J. Vreeland, Benny Johnson, Yoshikuni Kawaguchi, Steven H. Wei, Y. Nancy You, Eduardo Vilar, Jean-Nicolas Vauthey, Cathy Eng

**Affiliations:** 1 Department of Gastrointestinal Medical Oncology, The University of Texas MD Anderson Cancer Center, Houston, TX USA; 2 Department of Surgical Oncology, The University of Texas MD Anderson Cancer Center, Houston, TX USA; 3 Clinical Cancer Genetics Program, The University of Texas MD Anderson Cancer Center, Houston, TX USA; 4 Department of Clinical Cancer Prevention, The University of Texas MD Anderson Cancer Center, Houston, TX USA; 5 Present Address: Vanderbilt-Ingram Cancer Center, Nashville, TN USA

**Keywords:** Colorectal cancer, Oncogenes, Tumour biomarkers, Surgical oncology, Prognostic markers

## Abstract

**Background:**

The impact of molecular aberrations on survival after resection of colorectal liver metastases (CLM) in patients with early-age-onset (EOCRC) versus late-age-onset colorectal cancer (LOCRC) is unknown.

**Methods:**

Patients who underwent liver resection for CLM with known *RAS, BRAF* and MSI status were retrospectively studied. The prognostic impact of *RAS* mutations by age was analysed with age as a categorical variable and a continuous variable.

**Results:**

The study included 573 patients, 192 with EOCRC and 381 with LOCRC. The younger the age of onset of CRC, the greater the negative impact on overall survival of *RAS* mutations in the LOCRC, EOCRC, and ≤40 years (hazard ratio (HR), 1.64 (95% confidence interval (CI), 1.23–2.20), 2.03 (95% CI, 1.30–3.17), and 2.97 (95% CI, 1.44–6.14), respectively. Age-specific mortality risk and linear regression analysis also demonstrated that *RAS* mutations had a greater impact on survival in EOCRC than in LOCRC (slope: −4.07, 95% CI −8.10 to 0.04, *P* = 0.047, *R*^2^ = 0.08).

**Conclusion:**

Among patients undergoing CLM resection, *RAS* mutations have a greater negative influence on survival in patients with EOCRC, more so in patients ≤40 years, than in patients with LOCRC and should be considered as a prognostic factor in multidisciplinary treatment planning.

## Background

Colorectal cancer (CRC) is the third leading cause of cancer-related death in the US.^1^ Recently, CRC incidence and mortality rates have decreased significantly, likely because of greater utilisation of screening and increased therapeutic options.^1–3^ However, while the overall CRC incidence has declined, the incidence among individuals younger than 55 years has increased by ~2% per year.^4,5^ The reasons for the increase in CRC incidence in younger individuals are unclear. Lifestyle and environmental factors might explain the increasing incidence of early-age-onset colorectal cancer (EOCRC); obesity has been proposed as a main contributing factor; type 2 diabetes and changes in intestinal microbiome are suspected to be contributing factors.^5–10^

EOCRC exhibits marked molecular genetic heterogeneity, which must be considered in the analysis of risk factors, clinicopathological characteristics, prognostic biomarkers and potential therapeutic targets. The genomic landscapes of EOCRC and late-age-onset CRC (LOCRC) were characterised recently, and while many similarities were noted overall, marked differences were noted in rates of specific mutations when microsatellite stable (MSS) and high-frequency microsatellite instability (MSI-H) subgroups were analysed separately.^11^ Specifically, the most commonly mutated genes in MSS tumours, such as *APC*, *KRAS*, *BRAF*, *PIK3CA* and *AMER1*, were less frequent among EOCRC compared to LOCRC.^11^

Patients with EOCRC tend to be treated more aggressively than patients with LOCRC, with no definitive clinical evidence suggesting that EOCRC should be managed distinctly from LOCRC.^12–15^ Studies comparing the prognosis of patients with EOCRC versus LOCRC have shown conflicting results.^6^ Knowledge of the clinical relevance of common mutations according to the age of onset of CRC might be useful to develop distinct treatment strategies and predict the potential impact of personalised therapies in the increasing population of patients with EOCRC.

Hence, we opted to evaluate the prognostic impact of biomarkers starting with the impact of *RAS* mutations on the overall survival (OS) of patients with EOCRC and LOCRC who underwent curative-intent resection of colorectal liver metastases (CLM).

## Methods

### Study population

Patients who underwent resection of CLM with curative intent at The University of Texas MD Anderson Cancer Center from January 2006 to December 2016 with known *RAS, BRAF* and MSI status were retrospectively evaluated. Demographic, radiological, surgical, pathological, genetic and medical treatment characteristics were retrieved from electronic medical records. Patients who underwent liver-directed therapy (e.g., radiofrequency ablation, stereotactic radiation therapy) were excluded from the analysis. We followed the Reporting Recommendations for Tumour Marker Prognostic Studies (REMARK) guidelines.^16^ This study was approved by the Institutional Review Board of The University of Texas MD Anderson Cancer Center.

### Multidisciplinary management of CLM

Our institutional approach to surgical management of CLM has been previously detailed.^17^ CLM are deemed resectable when a hepatectomy can achieve a negative margin and preserve more than 30% of the standardised total liver volume.^18^ Patients with an anticipated insufficient future liver remnant are offered preoperative portal vein embolisation and staged hepatectomy. Patients are typically offered perioperative chemotherapy for a total of 12 cycles.^19^ After resection, patients are followed by either medical or surgical oncology with a history, physical examination, CEA and diagnostic imaging (CT chest, pelvis and abdomen with the liver protocol (2.5 mm) or an abdominal MRI) every 4 months for the first 2 years post liver resection and then every 6 months for the next 3 years.^20^

### Definitions

EOCRC was defined as CRC in patients <50 years old, and LOCRC was defined as CRC in patients ≥50 years old on the date of diagnosis. The primary tumour location was determined based on the surgical pathology report or on colonoscopy. Primary tumours located in the ascending colon or transverse colon were classified as right-sided tumours, and primary tumours located in the splenic flexure, descending colon, or rectum were classified as left-sided tumours. The extrahepatic disease was defined as preoperative radiological findings suggestive of metastatic disease outside the liver or intraoperative findings confirming extrahepatic disease. A positive surgical margin was defined as the presence of tumour cells <1 mm from the transection line. The primary tumour T category and N category were assigned according to the *AJCC Cancer Stating Manual*, eighth edition.^21^

### Somatic gene mutation profiling

*RAS* and *BRAF*^*V600E*^ mutational status were obtained from tissues of the primary tumour or metastatic site, and were assessed using PCR-based DNA sequencing analysis^22^ or next-generation sequencing^23^ performed on formalin-fixed, paraffin-embedded samples. Standardised testing for RAS mutational status was completed for exons 2 (codons 12 and 13), 3 (codons 59 and 61) and 4 (codons 117 and 146) of the *KRAS*, *NRAS* and *HRAS* genes, respectively. MSI status was determined using immunohistochemical analysis of formalin-fixed, paraffin-embedded tumour tissue.

### Statistical analysis

Descriptive statistics were used to summarise demographic and clinical data. Categorical variables were compared by chi-square and Fisher’s exact test. Continuous variables were compared by *t* test. Odds ratios (ORs) and 95% confidence intervals (CIs) were calculated with the use of the Baptista–Pike method and compared by Fisher’s exact test. Hazard ratios (HRs) and 95% CIs were calculated with the use of the Mantel–Haenszel method. Recurrence-free survival (RFS) was defined as the time in months from the date of the first liver resection to the date of radiological examination or medical evaluation (whichever had occurred first) conclusive of disease recurrence, regardless of the site. OS was defined as the time in months from the date of the first liver resection to the date of death from any cause. The patients who did not have documented recurrence or death or were lost to follow-up were censored on the date of the last contact. RFS and OS were estimated by the Kaplan–Meier method, and the survival curves were compared by the log-rank test. Age-specific mortality risk was estimated by dividing the number of patients in each age-at-diagnosis group who had died by the number of people in the same age group who were exposed to that risk (groups 20–29, 30–39, 40–49, 50–59, 60–69, 70–79 and ≥80 years old).

Prognostic factors were assessed by multivariate analysis using the Cox proportional hazards model, and *P* values < 0.05 were considered statistically significant. The backward stepwise elimination method was used in the variable selection in the regression model. Outliers were detected by using the Robust Regression Followed by the Outlier Identification method.^24^ Outliers were also defined as values greater than 1.5 times the interquartile 75.^25^ Linear regression was used to evaluate the association between continuous variables. The normality of the distribution of variables was examined by the Kolmogorov–Smirnov normality test. Analyses were performed by using SPSS 24.0 software (SPSS, Chicago, IL, USA) and GraphPad Prism software version 8.0.0 (GraphPad Software, San Diego, CA, USA).

## Results

### Study population

A total of 573 patients were eligible and were included in the study. All patients underwent at least one liver resection, 47 underwent two liver resections, 7 underwent three liver resections and 1 underwent four liver resections, for a total of 635 liver resections. The median age of the entire cohort was 54 years (range: 22–81 years). A total of 192 patients (34%) had EOCRC, and 381 patients (66%) had LOCRC. The EOCRC group had a higher proportion of women, left-sided tumours and extrahepatic disease compared to the LOCRC group (Table 1). Only a small minority of patients were determined to have a *BRAF* mutation or MSI-H tumour (<3%). The EOCRC and LOCRC groups did not differ statistically with respect to the frequency of *RAS* mutations, *BRAF* mutation and MSI-H tumours. Most patients received perioperative systemic chemotherapy. Preoperative and postoperative adjuvant chemotherapy were administered to 87 and 69% of the patients, respectively, in both the EOCRC and LOCRC cohorts. Perioperative systemic chemotherapy predominantly consisted of oxaliplatin-based regimens. Bevacizumab was used in preoperative chemotherapy in 61% of the patients. Of the 437 patients (76% of the entire cohort) who had pathologic response classification, 224 (51%) had a pathologic major response (<50% viable tumour cells), and 21 (5%) had a pathologic complete response. A positive surgical margin or margins in the liver resection specimen were found in 91 patients (16%).

**Table 1:** Baseline characteristics of the overall population by the timing of onset of colorectal cancer (*n* = 573).

Characteristic	Early-onset (*n* = 192)	Late-onset (*n* = 381)	*P* value
Median age at diagnosis (range), y	42 (22–49)	59 (50–81)	**<0.001**
*Sex*			
Male	99 (52)	236 (62)	**0.019**
Female	93 (48)	145 (38)	
RAS *status*			
Mutated	77 (40)	178 (47)	0.154
Wild-type	115 (60)	203 (53)	
BRAF *status*			
Mutated	5 (3)	4 (1)	0.294
Wild-type	163 (97)	293 (99)	
*MSI status*			
MSS	150 (98)	204 (97)	0.739
MSI-H	3 (2)	6 (3)	
*Tumour location*			
Ascending colon	29 (15)	93 (25)	**0.012**
Transverse colon	8 (4)	16 (4)	0.527
Descending colon	9 (5)	32 (8)	0.122
Rectosigmoid	146 (76)	240 (63)	**0.001**
*Sidedness*			
Right	37 (19)	109 (29)	**0.015**
Left	155 (81)	272 (71)	
*CEA level* *>* *10* *ng/mL*			
Yes	40 (22)	101 (27)	0.213
No	143 (78)	271 (73)	
*Bilobar disease*			
Yes	39 (21)	28 (26)	0.389
No	149 (79)	82 (75)	
*≥2 liver lesions*			
Yes	92 (48)	182 (49)	1
No	98 (52)	193 (51)	
*Pathologic response*			
<50% viable tumour cells	73 (46)	151 (54)	0.135
≥50% viable tumour cells	85 (54)	128 (46)	
*Pathologic complete response*			
Yes	11 (7)	10 (4)	0.16
No	147 (93)	269 (96)	
*Margin status*			
Positive	35 (18)	56 (15)	0.279
Negative	157 (82)	324 (85)	
*Extrahepatic disease*			
Yes	51 (27)	52 (14)	**<0.001**
No	141 (73)	329 (86)	
*Preoperative therapy*			
Yes	167 (87)	330 (87)	0.895
No	24 (13)	51 (13)	
*Preoperative bevacizumab*			
Yes	126 (66)	222 (58)	0.102
No	66 (34)	159 (42)	
*Postoperative therapy*			
Yes	125 (68)	260 (69)	1
No	58 (32)	119 (31)	

*CEA* carcinoembryonic antigen, *MSI* microsatellite instability, *MSI-H* high-frequency MSI, *MSS* microsatellite stable.

Values are presented as No. (%) unless otherwise indicated.

*p* < 0.05 was considered statistically significant.

### Association between *RAS* mutations and other baseline characteristics

An association between *RAS* mutations and sex was observed in the entire cohort and in the EOCRC and LOCRC subgroups (Supplementary Tables 1 and 2). In the entire cohort, *RAS* mutations were found in 54% (128/238) of women versus 38% (127/335) of men (OR: 1.90, 95% CI 1.36–2.65, *P* < 0.001). Similarly, an association between *RAS* mutations and sidedness was observed in the entire cohort and in the EOCRC and LOCRC subgroups. In the entire cohort, *RAS* mutations were found in 60% (87/146) of patients with right-sided tumours versus 39% (168/427) of patients with left-sided tumours (OR: 2.27, 95% CI 1.56–3.30, *P* < 0.001). An association between sex and sidedness was observed only in the LOCRC group, in which women had right-sided tumours more frequently than men did (36% vs. 24%, OR, 1.75, 95% CI 1.11–2.76, *P* = 0.019).

### Survival analysis

The median follow-up period after CLM resection was 70.1 months (95% CI, 64.5–75.7 months). Median OS was 70.0 months (95% CI, 61.9–78.2 months). A total of 271 deaths were observed (47%). Of the 470 patients who had the liver-limited disease, 366 (78%) had a recurrence. Median RFS was 11.4 months (95% CI, 10.4–12.3 months).

Multivariate analysis showed that four characteristics were associated with worse OS for all patients: *RAS* mutations, right-sided tumours, extrahepatic disease and lack of postoperative chemotherapy (Table 2). On multivariate analysis, specifically in the EOCRC group, the presence of extrahepatic disease had an impact on OS (*P* = 0.046); but sidedness and the provision of adjuvant chemotherapy had no bearing on outcome for OS (Table 3). In contrast in the LOCRC group, besides extrahepatic disease, sidedness and the use of adjuvant chemotherapy impacted OS (Table 4).

**Table 2:** Results of univariate and multivariate analysis of predictors for overall survival (OS) in the overall population (*n* = 573).

Characteristic	Categories	Univariate analysis			Multivariate analysis	
		Median OS (95% CI), mo	HR (95% CI)	*P* value	HR (95% CI)	*P* value
Age at diagnosis	<50 y (*n* = 192)	70.8 (55.5–86.1)	0.94 (0.73–1.23)	0.693	1.17 (0.77–1.77)	0.460
	≥50 y (*n* = 381)	68.6 (59.3–77.8)				
Sex	Male (*n* = 335)	75.5 (65.7–85.3 m)	**0.75 (0.59**–**0.95)**	**0.021**	0.88 (0.66–1.17)	0.393
	Female (*n* = 238)	58.9 (47.4–70.3)				
*RAS* status	Mutated (*n* = 255)	52.5 (45.2–59.7)	**1.74 (1.36–2.22)**	**<0.001**	**1.58 (1.23–2.03)**	**<0.001**
	Wild-type (*n* = 318)	78.4 (66.0–90.8)				
*BRAF* status	Mutated (*n* = 9)	47.2 (3.7–90.7)	1.32 (0.54–3.21)	0.536	2.01 (0.60–6.66)	0.252
	Wild-type (*n* = 456)	72.2 (63.4–81.0)				
MSI status	MSI-H (*n* = 9)	Not reached	0.32 (0.04–2.30)	0.259	0.28 (0.03–2.09)	0.218
	MSS (*n* = 354)	81.4 (67.8–95.0)				
Sidedness	Right (*n* = 146)	54.2 (47.7–60.7)	**1.63 (1.26–2.12)**	**<0.001**	**1.57 (1.20–2.05)**	**0.001**
	Left (*n* = 427)	76.3 (65.3–87.2)				
CEA level > 10 ng/mL	Yes (*n* = 141)	64.5 (54.1–75.0)	1.19 (0.90–1.56)	0.211	1.15 (0.72–1.83)	0.535
	No (*n* = 414)	72.0 (62.9–81.2)				
≥2 liver lesions	Yes (*n* = 263)	61.1 (50.3–71.8)	1.26 (0.99–1.60)	0.057	0.88 (0.58–1.33)	0.565
	No (*n* = 294)	77.9 (69.2–86.6)				
pCR	Yes (*n* = 21)	90.2 (40.4–139.9)	0.59 (0.28–1.27)	0.182	0.63 (0.28–1.43)	0.278
	No (*n* = 416)	67.3 (57.5–77.2)				
Margin status	Positive (*n* = 91)	66.6 (51.3–82.0)	0.89 (0.73–1.43)	0.894	1.16 (0.65–2.04)	0.607
	Negative (*n* = 481)	70.6 (61.4–79.8)				
Extrahepatic disease	Yes (*n* = 103)	46.0 (32.5–59.5)	**1.62 (1.21–2.17)**	**0.001**	**1.54 (1.15–2.07)**	**0.004**
	No (*n* = 470)	72.2 (63.7–80.7)				
Postoperative therapy	Yes (*n* = 385)	77.8 (67.6–88.0)	**0.66 (0.51–0.85)**	**0.001**	**0.66 (0.51–0.85)**	**0.001**
	No (*n* = 177)	57.1 (48.3–65.8)				

*CEA* carcinoembryonic antigen, *HR* hazard ratio, *MSI* microsatellite instability, *MSI-H* high-frequency MSI, *MSS* microsatellite stable, *pCR* pathologic complete response.

*p* < 0.05 was considered statistically significant.

**Table 3:** Results of univariate and multivariate analysis of predictors for overall survival (OS) in patients with EOCRC (*n* = 192).

Characteristic	Categories	Univariate analysis			Multivariate analysis	
		Median OS (95% CI), mo	HR (95% CI)	*P* value	HR (95% CI)	*P* value
Sex	Male (*n* = 99)	70.8 (48.8–92.8)	0.82 (0.53–1.27)	0.384	1.08 (0.60–1.94)	0.782
	Female (*n* = 93)	71.2 (48.8–92.8)				
*RAS* status	Mutated (*n* = 77)	53.3 (44.5–62.1)	**2.03 (1.30–3.17)**	**0.002**	**1.90 (1.20–3.02)**	**0.006**
	Wild-type (*n* = 115)	82.9 (52.3–113.4)				
*BRAF* status	Mutated (*n* = 5)	111.6 (0.0–∞)	1.22 (0.38–3.93)	0.730	1.18 (0.34–4.04)	0.784
	Wild-type (*n* = 163)	68.4 (53.7–83.2)				
MSI status	MSI-H (*n* = 3)	Not reached	0.04 (0.0–82.8)	0.424	0.0 (0.0–∞)	0.969
	MSS (*n* = 150)	72.0 (53.3–90.8)				
Sidedness	Right (*n* = 37)	47.8 (24.8–70.8)	**1.84 (1.11–3.02)**	**0.016**	1.71 (0.99–2.94)	0.050
	Left (*n* = 155)	81.7 (58.5–105.0)				
CEA level > 10 ng/mL	Yes (*n* = 40)	71.2 (41.9–100.5)	1.10 (0.65–1.87)	0.698	1.23 (0.62–2.45)	0.544
	No (*n* = 143)	68.4 (53.0–83.8)				
≥2 liver lesions	Yes (*n* = 83)	56.4 (39.4–73.4)	1.48 (0.95–2.30)	0.077	**1.60 (1.02–2.49)**	**0.038**
	No (*n* = 102)	72.2 (52.5–91.9)				
pCR	Yes (*n* = 11)	90.2 (0.0–∞)	0.63 (0.19–2.01)	0.438	0.57 (0.13–2.41)	0.446
	No (*n* = 147)	70.8 (54.8–86.8)				
Margin status	Positive (*n* = 35)	72.2 (0.0–∞)	0.85 (0.48–1.52)	0.597	1.06 (0.53–2.12)	0.856
	Negative (*n* = 157)	68.4 (51.8–85.1)				
Extrahepatic disease	Yes (*n* = 51)	53.3 (33.1–73.5)	**1.76 (1.11–2.80)**	**0.015**	**1.64 (1.00–2.66)**	**0.046**
	No (*n* = 141)	72.2 (60.2–84.2)				
Postoperative therapy	Yes (*n* = 125)	72.2 (44.0–100.4)	0.71 (0.44–1.12)	0.144	0.64 (0.40–1.02)	0.064
	No (*n* = 58)	59.5 (43.8–75.1)				

*CEA* carcinoembryonic antigen, *HR* hazard ratio, *MSI* microsatellite instability, *MSI-H* high-frequency MSI, *MSS* microsatellite stable, *pCR* pathologic complete response.

*p* < 0.05 was considered statistically significant.

**Table 4:** Results of univariate and multivariate analysis of predictors for overall survival (OS) in patients with LOCRC (*n* = 381).

Characteristic	Categories	Univariate analysis			Multivariate analysis	
		Median OS (95% CI), mo	HR (95% CI)	*P* value	HR (95% CI)	*P* value
Sex	Male (*n* = 236)	75.5 (64.2–86.8)	**0.70 (0.52–0.94)**	**0.019**	0.90 (0.63–1.30)	0.591
	Female (*n* = 145)	56.8 (43.2–70.4)				
*RAS* status	Mutated (*n* = 178)	52.2 (39.0–65.4)	**1.64 (1.23–2.20)**	**0.001**	**1.53 (1.13–2.05)**	**0.005**
	Wild-type (*n* = 203)	78.3 (62.8–93.9)				
*BRAF* status	Mutated (*n* = 4)	47.2 (4.6–89.8)	1.44 (0.35–5.85)	0.605	1.98 (0.47–8.24)	0.345
	Wild-type (*n* = 293)	78.2 (68.3–88.1)				
MSI status	MSI-H (*n* = 6)	Not reached	0.59 (0.08–4.26)	0.603	1.12 (0.12–9.74)	0.916
	MSS (*n* = 204)	83.2 (69.2–97.3)				
Sidedness	Right (*n* = 109)	54.3 (47.1–61.6)	**1.56 (1.15–2.12)**	**0.004**	**1.53 (1.12–2.09)**	**0.007**
	Left (*n* = 272)	76.3 (64.5–88.1)				
CEA level > 10 ng/mL	Yes (*n* = 101)	62.6 (52.7–72.5)	1.22 (0.88–1.69)	0.215	1.19 (0.86–1.66)	0.286
	No (*n* = 271)	74.6 (64.2–84.9)				
≥2 liver lesions	Yes (*n* = 180)	63.5 (49.8–77.1)	1.18 (0.88–1.57)	0.256	1.01 (0.71–1.44)	0.937
	No (*n* = 192)	78.2 (66.6–89.9)				
pCR	Yes (*n* = 10)	93.6 (0.0–∞)	0.58 (0.21–1.58)	0.291	0.80 (0.25–2.58)	0.713
	No (*n* = 269)	64.9 (51.7–78.1)				
Margin	Positive (*n* = 56)	62.6 (47.7–77.5)	1.15 (0.75–1.74)	0.509	1.02 (0.56–1.86)	0.944
	Negative (*n* = 324)	72.1 (61.9–82.3)				
Extrahepatic disease	Yes (*n* = 52)	45.1 (33.3–56.9)	**1.57 (1.07–2.32)**	**0.021**	**1.52 (1.03–2.25)**	**0.033**
	No (*n* = 329)	72.1 (62.3–82.0)				
Postoperative therapy	Yes (*n* = 260)	78.2 (66.7–89.7)	**0.64 (0.47–0.86)**	**0.004**	**0.66 (0.48–0.89)**	**0.008**
	No (*n* = 119)	56.8 (46.1–67.5)				

*CEA* carcinoembryonic antigen, *HR* hazard ratio, *MSI* microsatellite instability, *MSI-H* high-frequency MSI, *MSS* microsatellite stable, *pCR* pathologic complete response.

*p* < 0.05 was considered statistically significant.

**Fig. 1:**
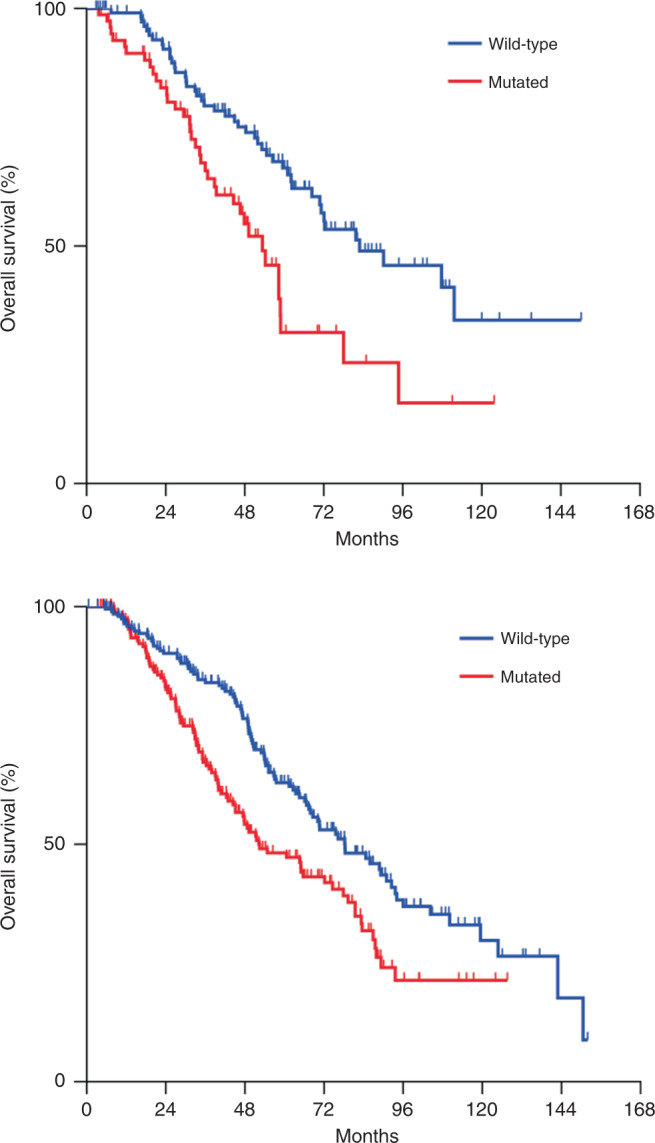
Overall survival by *RAS* mutation status. Overall survival by RAS mutation status in patients with (**a**) early-age-onset (*n* = 192) and (**b**) late-age-onset (*n* = 381) colorectal cancer.

**Fig. 2:**
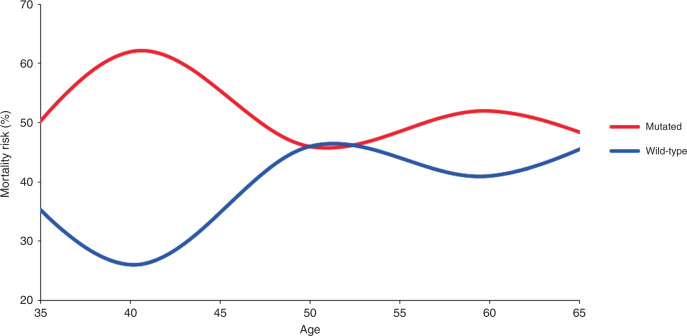
Age-specific mortality risk by RAS status. Age-specific mortality risk was estimated by dividing the number of patients in each age-at-diagnosis group who had died by the number of people in the same age group who were exposed to that risk (groups 20–29, 30–39, 40–49, 50–59, 60–69, 70–79 and ≥80 years of age). The area between the curves is the sum of the hazard ratios of death; the larger the area, the stronger the age-specific prognostic value of RAS mutations. RAS mutations had a stronger prognostic value in early-onset CRC. The fact that the curves are closer around 50 years suggests that the risk is similar in patients diagnosed with CRC in middle age.

Overall, patients who harboured *RAS* mutations had a 58% higher risk of death following liver resection (Table 2). The negative prognostic impact of *RAS* mutations was greater in the EOCRC group than in the LOCRC group (HR: 1.90, 95% CI 1.20–3.02, *P* = 0.006 versus HR: 1.53, 95% CI 1.13–2.05, *P* = 0.005) (Tables 3 and 4; Fig. 1a, b). Also, the negative prognostic impact of *RAS* mutations was greater in the population of patients with age at onset of CRC ≤ 40 years (*n* = 80) (HR: 2.97, 95% CI 1.44–6.14, *P* < 0.05) than in patients in the entire EOCRC group or the LOCRC group. Furthermore, when we analysed age as a continuous variable by age-specific mortality risk and by linear regression, our data suggested that *RAS* mutations had a higher impact on OS in the EOCRC group than in the LOCRC group (Fig. 2 and Supplementary Figs. 1 and 2). Analysis by residual plot raised the possibility that this age-dependent association of *RAS* mutations might be influenced by the presence of outliers (not shown).

Our cohort had a small number of patients in the extremely young and old age groups (<35 years and >70 years), which may explain the presence of possible HR outliers. By applying the Robust Regression Followed by Outlier Identification method for the detection of outliers, we identified 10 groups of age with HRs as outliers (ages 26, 31, 32, 36, 39, 45, 69, 70, 72 and 74 years), which included 58 patients (10% of the population; in these age groups, patients with *RAS* mutations had a risk of death much higher than the risk of death of the remaining age groups). Similarly, identifying outliers as values greater than 1.5 times the interquartile 75 (HR > 7.12) produced the same result. Comparison of the characteristics of the 23 patients with *RAS* mutations in the outlier group with the 232 patients with *RAS* mutations in the remaining population showed that the outliers had greater rates of extrahepatic disease (26% versus 20%) and lower rates of receipt of adjuvant chemotherapy (56% versus 66%) and neoadjuvant chemotherapy (74% versus 86%), which are poor prognostic factors. However, the difference between the outliers and the other patients did not reach statistical significance for any of these comparisons. Multivariate analysis for OS of the outliers confirmed that patients with *RAS* mutations had a higher risk of death (HR: 16.8, 95% CI 5.7–48.8, *P* < 0.001). Age and sidedness were not prognostic factors among the outliers (*P* > 0.05).

A multivariate analysis of the entire cohort after exclusion of the outliers (*n* = 515) demonstrated that *RAS* mutations remained a prognostic factor (HR: 1.51, 95% CI 1.17–1.95, *P* = 0.002), together with the extrahepatic disease (HR: 1.60, 95% CI 1.18–2.18, *P* = 0.003) and carcinoembryonic antigen level ≥10 ng/mL (HR: 1.39, 95% CI 1.04–1.85, *P* = 0.024).

A multivariate analysis of the EOCRC patients showed that *RAS* mutations, extrahepatic disease, and ≥2 liver lesions were associated with worse OS (Table 3). Compared with the subgroup of patients with EOCRC and wild-type *RAS*, the subgroup with EOCRC and *RAS* mutations (*n* = 77) had higher proportions of women, right-sided tumours and extrahepatic disease (Supplementary Table 1). A multivariate analysis of the LOCRC patients showed that *RAS* mutations, right-sided tumours, extrahepatic disease and lack of postoperative chemotherapy were associated with worse OS (Table 4). Since patients with age near 50 years represent a meaningful subgroup, and their tumours might share molecular characteristics, which could compromise the survival analysis, we repeated the univariate and multivariate analyses excluding patients aged 46 to 59 years. The results were similar to those for the entire cohort. Age group (≤45 years and ≥60 years) was not associated with OS, whereas *RAS* mutations were a prognostic factor (HR: 1.96, 95% CI 1.38–2.79, *P* < 0.001).

## Discussion

The findings of our study suggest that the prognostic value of *RAS* mutations in patients who underwent CLM resection differs according to the age of onset of CRC and has a greater influence on survival in patients with EOCRC, especially if diagnosed ≤40 years of age.

Our analysis has several positive aspects. The retrospective design of our single-institution study provides a large and homogeneous population of CRC patients who were uniformly deemed to have surgically resectable CLM disease (100% of the patients had colorectal cancer with liver metastases who underwent surgical resection for curative intent) which allows us to review the role of these biomarkers on the prognosis of both EOCRC and LOCRC. Patients who underwent liver-directed therapy were excluded from this analysis to further support the homogeneity of the patient population. There was the uniformity of the use of neoadjuvant and adjuvant chemotherapy in both groups. Anti-VEGF therapy was the only biologic provided in the neoadjuvant setting. Furthermore, practice patterns at our institution reserved anti-EGFR therapy for the refractory setting especially given the concerning findings of worse PFS when utilised in the neoadjuvant setting.^26^ The long-term median follow-up of 70.1 months also demonstrates adequate follow-up followed by the uniformity of surveillance for all resected patients.

Our study does have its limitations. This is a single-institution, retrospective analysis. The median age of our patient population was younger than the average age of patient in the US impacted by colorectal cancer and is a reflection of the young patient population that is commonly referred to as academic institutions. The primary molecular markers evaluated in this analysis were limited to *RAS*, *BRAF* and MSI because these were accepted molecular markers of interest at that time based on published or recently presented data.^27–29^ Our single-institution study was not powered to differentiate the impact of rare, poor prognostic, specific *RAS* mutations (e.g., NRAS). Cercek et al. previously reported patients with the *NRAS* MT were less likely to proceed with surgical resection and had reduced OS relative to *KRAS* MT patients.^30^

Our findings may be influenced by a subgroup of patients with *RAS* mutations who had a higher risk of death than patients with *RAS* mutations in the remaining population. These outliers were identified by two concordant methods. These patients accounted for 10% of the patients in the entire cohort and had increased rates of poor prognostic factors in addition to *RAS* mutations. This raises the hypothesis that there is a subgroup of patients with *RAS* mutations who have an extremely high risk of death after CLM resection. The absence of statistically significant differences in the rates of poor prognostic factors between the outliers with *RAS* mutations and the patients with *RAS* mutations in the remaining population was probably due to the small sample size of outliers. The lower frequency of patients in the extremely young and old age groups might explain the presence of the outliers. However, a definitive explanation would only be possible with an analysis of a much larger population of patients in those age groups (<35 years and >70 years).

Since the use of postoperative therapy was not mandatory and it was a significant predictor for OS on the overall population on multivariate analysis, imbalances in the rates of postoperative therapy among subgroups could influence our findings. However, there was no difference in the use of postoperative therapy by age and by RAS status (Supplementary Tables 1 and 2).

Our findings regarding the prognostic value of *RAS* mutations in patients with EOCRC are concordant with previous reports of lower tumour mutational burden in this patient group. Tumour mutational burden increases significantly with age and maybe up to 2.4 times as high in someone diagnosed with cancer at 90 years of age as it is in someone diagnosed with cancer at 10 years of age.^31^ We were able to find no similar studies examining other *RAS*-driven malignancies, such as pancreatic cancer and non-small cell lung cancer (NSCLC), possibly because few young patients experience these cancers. However, a study in a cohort of NSCLC patients demonstrated that patients younger than 50 years had a higher probability of harbouring a targetable genotype.^32^ This suggests that age might be an underappreciated marker for specific mutational profiles. Interestingly, we found no such association of age with *BRAF* mutation or MSI-H. This is most likely because our cohort had very low rates of these two molecular abnormalities.

The development of tailored therapies for *RAS*-driven malignancies has been elusive. A recently presented Phase 1 trial showed encouraging results of targeted therapies for the specific *KRAS*^*G12C*^ mutation.^33^ The study evaluated the efficacy and safety of a *RAS*^*G12C*^ inhibitor (AMG 510) in patients who had locally advanced or metastatic solid tumours and harboured *RAS*^*G12C*^ mutation. Of ten patients with NSCLC, five patients showed a partial response, and four had stable disease. These findings had a minimal benefit in the colorectal cohort.^34,35^

Our study suggests that *RAS* mutations have a significantly greater impact on survival after CLM resection in patients with EOCRC than in patients with LOCRC. In addition, in the presence of extrahepatic disease, consideration of liver resection in an EOCRC patient should be considered with caution. With next-generation sequencing (NGS) now as a standard of care, it is likely additional molecular markers will be identified in the EOCRC versus LOCRC patient population that may be prognostic and possibly predictive for OS following CLM resections. Inclusion of adjunct tools, such as immunoscore,^36^ a robust and validated test of the host immune reaction measuring CD3+ and CD8+ T-cell densities within the tumour and/or detection of ctDNA may be considered for determination of the risk of recurrence.^37,38^ To our knowledge, this is the first study determining the influence of *RAS* mutations on overall survival from the perspective of the age of onset in *RAS*-driven colorectal cancer liver resections.

## Supplementary information

Supplementary file

## Data Availability

The datasets of the study contain personal health information and are confidential. These are available to readers upon reasonable request.
